# A Study on the Diagnostic Approach Using Real-Time Video Capsule Endoscopy in Dogs with Acute Vomiting

**DOI:** 10.3390/ani15071056

**Published:** 2025-04-05

**Authors:** Hyomi Jang, Young Joo Kim, Dong-In Jung

**Affiliations:** 1 VIP Animal Medical Center (Cheongdam), Seoul 06068, Republic of Korea; hm100su@gmail.com; 2 College of Veterinary Medicine, Western University of Health Sciences, Pomona, CA 91766, USA; 3 College of Veterinary Medicine, Gyeongsang National University, Jinju 52828, Republic of Korea

**Keywords:** capsule endoscopy, dog, gastric foreign body, real time, vomiting, gastritis

## Abstract

This study aimed to evaluate the efficiency and sensitivity of real-time video capsule endoscopy (RT-VCE) when detecting surgical and nonsurgical gastric lesions in dogs that presented with acute vomiting. Additionally, the patient tolerance and the utility of the RT-VCE data for clinical decision-making were assessed. Eleven client-owned dogs with an acute onset of vomiting were included. Each dog received an antiemetic and antacid before undergoing an RT-VCE (MC1200, MiroCam^®^, Intromedic, Seoul, Republic of Korea). We independently reviewed the RT-VCE images to assess the image quality, detect gastric lesions, and make clinical decisions. The time taken to reach a diagnosis and the inter-clinician agreement were evaluated. RT-VCEs were successfully completed in all the dogs, with a mean time to diagnosis of 21.82 ± 15.26 min (range: 1–48 min). Both clinicians reached unanimous agreement on the RT-VCE findings. Five dogs were diagnosed with gastric foreign bodies and underwent surgical removal, while the remaining six were diagnosed with gastritis, a gastric ulcer or erosion, or hemorrhages, which were managed medically. No adverse effects, including vomiting, were observed after the capsule administration. RT-VCE proved to be an effective, accurate, and well-tolerated diagnostic method for evaluating gastric conditions in dogs with acute vomiting. Its rapid diagnostic capability and non-invasive nature make it a valuable modality in emergency veterinary settings.

## Simple Summary

RT-VCE is a non-invasive imaging method that enables visualization of the gastrointestinal tract. In this study, its application was focused on the stomach in dogs with acute vomiting to detect relevant abnormalities. This study evaluated the effectiveness, accuracy, and safety of RT-VCE in diagnosing gastric lesions to distinguish between cases that required surgery and those treatable with medication. Eleven dogs that presented with acute vomiting underwent RT-VCE examinations, which allowed veterinarians to quickly identify gastric foreign bodies that required surgical removal and conditions like gastritis or an ulcer or erosion that were treatable medically. RT-VCE provided rapid and reliable results without any adverse effects, which proved to be highly beneficial in veterinary emergency care.

## 1. Introduction

In veterinary medicine, the accurate and timely diagnosis of gastrointestinal disorders in dogs is crucial for effective treatment and improved patient outcomes [1,2,3,4]. Among the common clinical presentations, acute vomiting in dogs often necessitates a prompt investigation to identify the underlying cause and guide appropriate therapeutic interventions [5].

Traditional diagnostic modalities for acute vomiting in dogs—physical examination, laboratory diagnostics, radiography, and ultrasonography—offer crucial insights. However, their sensitivity may be inadequate for detecting subtle or early-stage abnormalities in the GI tract [1,5,6,7,8].

Over the past few decades, the field of veterinary medicine has seen significant advancements in endoscopic techniques [9]. Conventional endoscopy, which involves the insertion of a flexible or rigid endoscope into the gastrointestinal tract, has been widely utilized for diagnosing gastrointestinal disorders and has provided valuable insights into the gastrointestinal tract in small animals [9,10]. However, the limitations of conventional endoscopy, such as incomplete visualization of the entire gastrointestinal tract and the requirement for sedation or general anesthesia, have prompted the exploration of alternative approaches to enhance its diagnostic capabilities.

In recent years, advancements in medical imaging technology have led to the emergence of a novel diagnostic method known as video capsule endoscopy, which offers a non-invasive and comprehensive approach for examining the gastrointestinal system [11,12,13,14,15].

Importantly, RT-VCE has emerged as a significant advancement in both human and veterinary endoscopy, allowing for instantaneous visualization of the gastrointestinal mucosa spanning from the esophagus to the rectum [11,14]. This technology involves a wireless capsule equipped with a miniature camera that transmits images to an external receiver unit worn by the patient. As the capsule traverses the gastrointestinal tract, it captures high-resolution images, providing a detailed examination of the mucosal surfaces and enabling the identification of abnormalities. Unlike conventional endoscopy, RT-VCE eliminates the need for anesthesia or sedation, reduces potential risks, and increases patient comfort [11,14].

Despite these advantages and its success in human gastroenterology, the potential of RT-VCE in veterinary practice, particularly for diagnosing acute vomiting in dogs, has not been widely adopted or investigated for this indication in veterinary medicine. The application of RT-VCE in an emergency veterinary setting could hold the key to a more comprehensive understanding of the canine GI tract, enabling the identification of both surgical and nonsurgical gastric lesions with increased sensitivity.

This study aimed to investigate the diagnostic accuracy and efficiency of RT-VCE in identifying surgical or nonsurgical gastric lesions in dogs that presented with acute vomiting. Additionally, this study assessed the patient acceptance and the ability of clinicians to make informed decisions based on the data obtained from RT-VCEs. By evaluating the performance of this innovative diagnostic modality, we can expand our understanding of its potential applications in emergency veterinary settings and contribute to the advancement of diagnostic techniques in veterinary clinics.

## 2. Case Description

This case study included 11 client-owned dogs (mean age 6.2 ± 4.7 years, mean weight 21.14 ± 10.84 kg), as outlined in Table 1. These subjects presented as emergency cases at emergency animal medical centers due to the recent onset of acute vomiting within a few days prior to admission. Despite an in-depth clinical history, physical examination, blood tests, and basic thoracic and abdominal radiographic evaluations, accurately determining the underlying etiology of the acute vomiting remained challenging.

**Figure 1:**
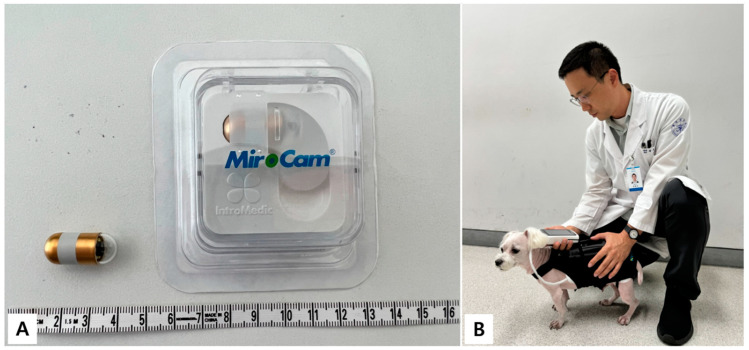
Capsule endoscope used in this study (**A**). During RT-VCE examination, veterinarians interpreted with real-time viewer (**B**).

Given the reluctance of clients for invasive diagnostic methods and time-consuming procedures, we recommended capsule endoscopy as a less invasive and anesthesia-free alternative for diagnostic evaluation. After obtaining informed consent from the owners, we proceeded with the RT-VCE examination using the MiroCam^®^ (MC1200, Intromedic, Seoul, Republic of Koea) system (Figure 1A).

Prior to the capsule endoscopy, the dogs were administered an antiemetic medication (maropitant citrate, 1 mg/kg, subcutaneously). As the dogs presented with acute vomiting, an antiemetic was administered prior to the capsule endoscopy to reduce the likelihood of vomiting during the procedure, which could interfere with the capsule passage or result in premature expulsion. Maropitant citrate was selected due to its well-established antiemetic efficacy, favorable safety profile, and rapid onset of action in canine patients. A notable advantage of the MiroCam^®^ system is its ability to monitor video images on an external screen in real time.

The RT-VCE images were reviewed and interpreted by veterinarians directly from a real-time display (Figure 1B). All procedures that involved dogs complied with the guidelines approved by the IACUC of Gyeongsang National University (GNU-240325-D0067).

Among the 11 dogs included in this study, seven were spayed females and four were castrated males. The median age of the dogs was 6.2 years, which ranged from 0.5 to 14 years. Six dogs were older than 6 years of age. The median weight was 21.82 kg, with a range of 5.5 to 37.64 kg. This study included diverse breeds, such as a Boston Terrier and a Golden Retriever. The specific breeds included a Pitbull Terrier (two dogs), Labrador Retriever, Golden Retriever, Golden Doodle, Shepherd Mix, Boxer, Yorkshire Terrier Mix, Shih Tzu, Wheaten Terrier, and Boston Terrier. All 11 dogs presented with symptoms of acute vomiting, with two instances of hematemesis and one occurrence of concurrent diarrhea. The detailed data are presented in Table 1.

All the dogs showed no abnormal findings related to acute vomiting in their blood test results and thoracic radiographs. Abdominal ultrasonography was performed in three of the eleven dogs, but the findings were either non-specific or inconclusive regarding the gastric contents. Although ultrasound is clearly a useful modality for identifying gastric foreign bodies or other possible causes of vomiting, it was not performed in all cases due to various limitations, such as the cost considerations or owner refusal. Abdominal radiographs showed no specific findings in most cases. In some dogs, radiopaque shadows within the stomach suggested possible foreign bodies; however, accurate determination was difficult. One dog presented with radiographic findings suggestive of a round-shaped foreign body within the stomach, suspected to be a ball.

**Figure 2:**
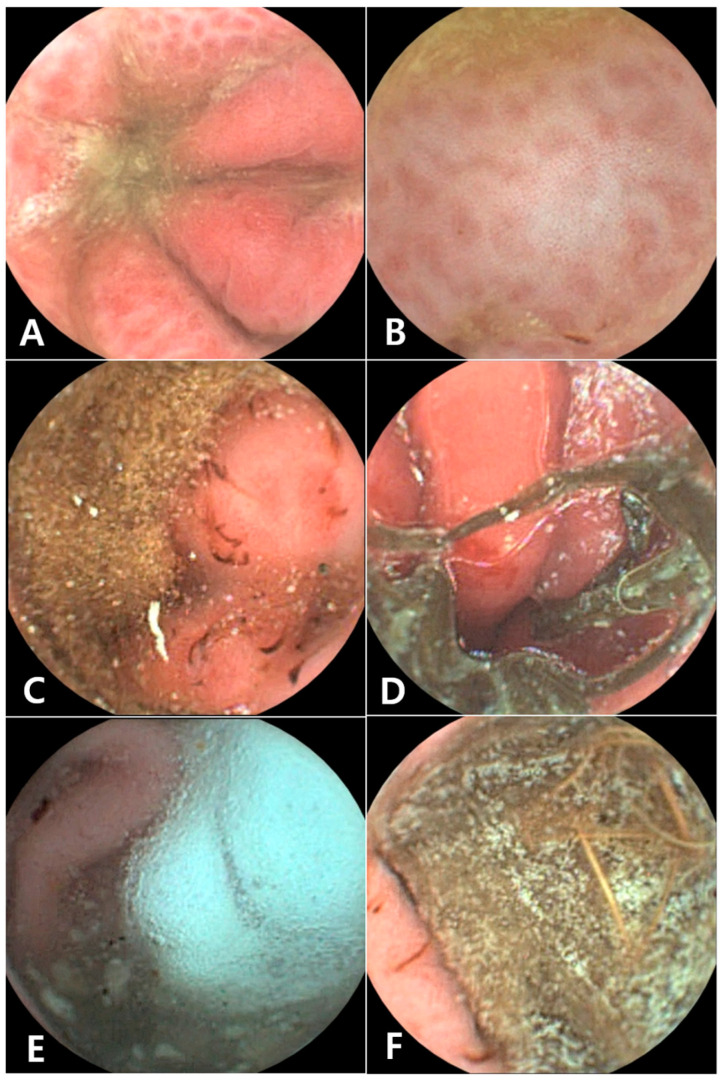
RT-VCE-captured images of the patients in this study. (**A**,**B**) show severe redness and an ulcer or erosion of the gastric mucosa. In panels (**C**,**D**), sand (**C**) and grass (**D**) are visible in the stomach, and bleeding due to inflammation of the gastric mucosa is observed. In (**E**) (sylofoam) and (**F**) (brush), foreign bodies are observed in the stomach.

**Figure 3:**
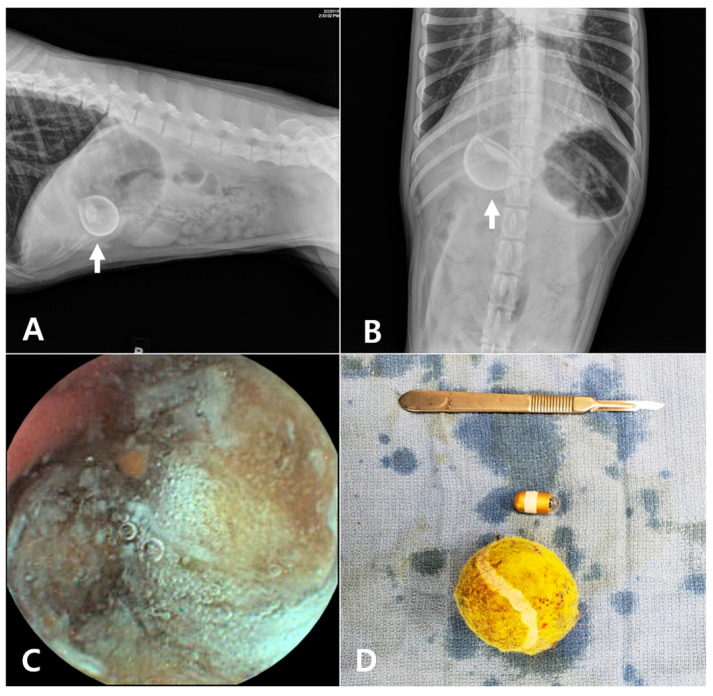
In case no. 3, abdominal radiographs revealed a round-shaped foreign body in the stomach ((**A**,**B**): arrow). The capsule endoscopy identified the foreign body as a suspected tennis ball (**C**), which was subsequently removed surgically (**D**).

**Figure 4:**
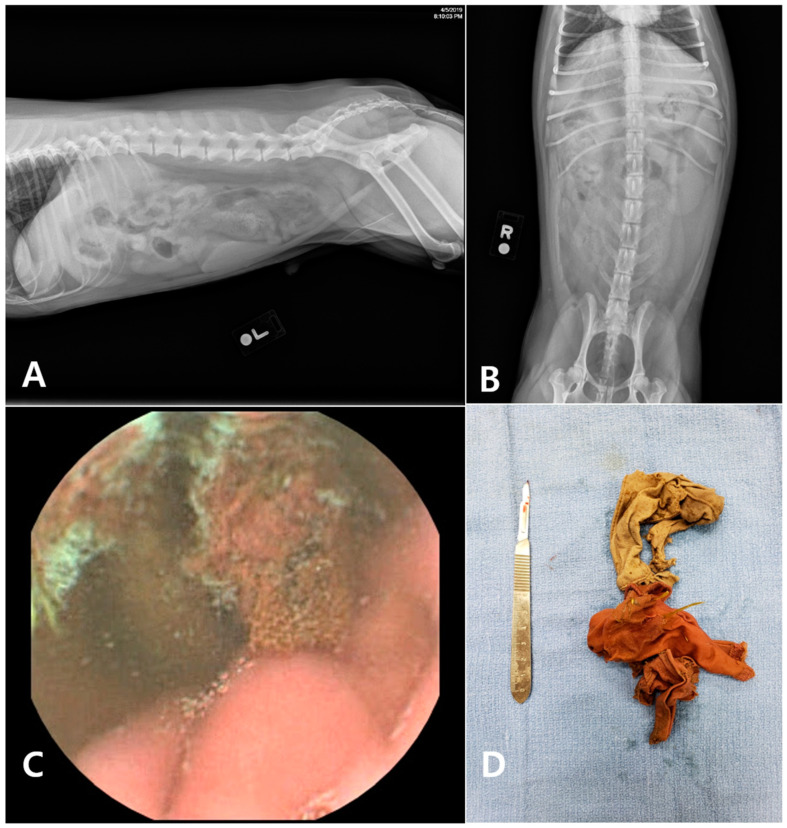
In case no. 5, the abdominal radiographs revealed no remarkable finding (**A: left lateral view**, **B: dorsal view**). Fabric-like foreign bodies were identified in the stomach during the capsule endoscopy (**C**). These foreign bodies (underwear) were surgically removed (**D**).

The post-RT-VCE examination revealed the etiology of the acute vomiting in all 11 dogs. There was no discrepancy in the diagnoses between the veterinarians, and the time to reach a medical decision ranged from 1 to 48 min (mean 21.82 ± 15.26 min). Five dogs were diagnosed with gastric foreign bodies. Five dogs were diagnosed with gastritis, and one dog was diagnosed with a gastric ulcer or erosion (Figure 2). The five dogs diagnosed with acute vomiting secondary to gastric foreign bodies underwent surgical removal of the object (Figure 3 and Figure 4).

The remaining six dogs diagnosed with gastritis received medical management, including fluid therapy, the administration of proton pump inhibitors (omeprazole; 1 mg/kg PO BID), and dietary modifications (hypoallergenic diet). None of the dogs vomited from the capsule endoscopes after the procedure and no other adverse effects were observed. The RT-VCE diagnostic-related data are summarized in Table 2.

## 3. Discussion

The present study demonstrated that RT-VCE is a promising method for the diagnostic assessment of acute vomiting in dogs. Notably, this study revealed that RT-VCE provided a swift and efficient means of identifying both surgical and nonsurgical gastric lesions, potentially offering a rapid route for decision-making in an emergency veterinary setting.

Gastric foreign bodies, gastritis, and gastric ulceration are common causes of acute vomiting in dogs [5,6,8]. Delays in diagnosis and treatment can result in serious consequences, including progression to more severe disease states, complications, or even death [2,3,4,5]. Traditional diagnostic modalities, such as a physical examination, laboratory diagnostics, radiography, and ultrasonography, play crucial roles in the diagnostic workup of acute vomiting in dogs [5,6,8]. Nevertheless, these approaches may have limitations in their ability to detect subtle or early-stage abnormalities within the GI tract [5,6,7,16]. In this study, abdominal ultrasonography could have provided additional diagnostic information in patients suspected of having gastric foreign bodies; however, precise comparisons were difficult because ultrasonography was not performed in all patients included in this case study.

Although valuable, conventional endoscopy is limited by its inability to visualize the entire gastrointestinal tract and the need for sedation or general anesthesia [9,10]. RT-VCE circumvents these limitations by offering a noninvasive, anesthesia-free approach that provides a comprehensive view of the GI mucosa from the esophagus to the rectum.

Our study highlighted several advantages of RT-VCE for evaluating acute vomiting in dogs. First, RT-VCE enables the real-time visualization of the GI mucosa, supporting prompt decision-making in emergency situations. In our study, the time to reach a medical decision ranged from 1 to 48 min, with a mean time of 21.82 ± 15.26 min. This rapid diagnostic process is invaluable in an emergency setting, where timely intervention can substantially improve patient outcomes. This ability to deliver rapid diagnostic insights not only facilitates early intervention but also streamlines triage and clinical prioritization in emergency veterinary care. Especially in patients with unstable conditions, minimizing the time to decision-making can be critical in preventing clinical deterioration.

Second, RT-VCE offers excellent image quality, allowing for the identification of both surgical and non-surgical gastric lesions. In our study, five dogs were diagnosed with gastric foreign bodies that required surgical intervention for removal. The high-resolution images provided by RT-VCE facilitated the identification of foreign objects, which enabled timely surgical intervention. Additionally, RT-VCE allowed for the diagnosis of gastritis and gastric ulceration. Furthermore, in cases of complete obstruction not caused by a foreign body, the capsule may experience significant delays in excretion or may not pass naturally. Nevertheless, one of the notable advantages of RT-VCE is that it enables real-time, anesthesia-free visualization of the obstructed region, providing critical information about the location, shape, and likely etiology of the obstruction. This feature can guide clinical decision-making and help identify the most appropriate surgical approach, as the capsule’s point of retention typically corresponds to the obstructive lesion. Although the diagnostic value of RT-VCE may be limited when a foreign body is clearly identified on abdominal radiography, as seen in case 3, this modality still offers important clinical benefits. RT-VCE allows for direct visualization of the gastric mucosa and any concurrent inflammatory lesions prior to anesthesia or surgical intervention. Additionally, it helps evaluate the type and condition of the foreign object, supporting clinicians in determining whether endoscopic removal is feasible or whether surgical intervention is more appropriate.

Third, RT-VCE demonstrated good patient acceptance and tolerability. None of the dogs in this study vomited the capsule endoscope after the procedure, and no adverse effects were observed. Because RT-VCE eliminates the need for anesthesia or sedation, it reduces the risks associated with these interventions, thereby enhancing patient safety and comfort.

The unanimous interpretive agreement between the two veterinary clinicians in our study suggests that the RT-VCE images are reliable and interpretable, aiding in diagnostic consistency. This interpretive agreement is vital in emergency settings, where discrepancies in diagnoses can lead to delays in treatment or inappropriate interventions.

The limitations of this study include the small sample size, and the lack of comparative analysis with conventional endoscopy and abdominal ultrasound. Additionally, long-term outcomes of the diagnosed cases were not assessed in this study. Future studies with larger sample sizes, comparative analyses, and long-term follow-up assessments are required to establish the clinical significance and utility of RT-VCE in the diagnostic workup for acute vomiting in dogs.

## 4. Conclusions

In conclusion, RT-VCE is a valuable diagnostic method for the evaluation of acute vomiting in dogs. This study suggests that RT-VCE could provide a fast, accurate, and well-tolerated method for diagnosing and managing acute gastric conditions in dogs. Its ability to provide real-time, high-quality images of the GI mucosa enabled rapid identification of both surgical and nonsurgical gastric lesions, which supported prompt decision-making in an emergency veterinary setting. These findings could revolutionize the standard practice of veterinary medicine and improve patient outcomes.

## Figures and Tables

**Table 1:** Summary of signalment data and clinical signs.

No.	Sex	Breed	Age (Yrs)	BW (Kg)	Clinical Signs	Radiographic Findings
1	SF	Boxer	2.92	28.18	Acute vomiting (hematemesis)	NRF
2	SF	Labrador Retriever	0.5	22.82	Acute vomiting	NRF
3	SF	Shepherd Mix	2	29.09	Acute vomiting	A round-shaped foreign body suspected in the stomach
4	SF	Pitbull Terrier	9	28.36	Acute vomiting	NRF
5	CM	Yorkshire Terrier Mix	2	9.50	Acute vomiting	NRF
6	SF	Wheaten Terrier	6.58	10.91	Acute vomiting	Foreign body suspected in the stomach
7	SF	Pitbull Terrier	9.17	28.36	Acute vomiting	NRF
8	CM	Golden Retriever	11	37.64	Acute vomiting	NRF
9	CM	Golden Doodle	1	24.27	Acute vomiting	Foreign body suspected in the stomach
10	CM	Shih Tzu	14	7.5	Acute vomiting (hematemesis)	NRF
11	SF	Boston Terrier	10	5.5	Acute vomiting, diarrhea	NRF

SF: spayed female, CM: castrated male, NRF: no remarkable finding.

**Table 2:** Summary of RT-VCE diagnostic-related data and medical decision results.

No.	Medical Decision with RT-VCE	Time to Medical Decision (min)	Therapeutic Approaches After Diagnosis
1	Gastritis (ulcer or erosion, hemorrhage)	48	Medical management
2	Foreign body (leather piece, stylofoam)	33	Surgery
3	Foreign body (tennis ball)	13	Surgery
4	Gastritis (hemorrhage)	12	Medical management
5	Foreign body (pieces of underwear)	1	Surgery
6	Foreign body (brush)	3	Surgery
7	Gastric ulcer or erosion	30	Medical management
8	Gastritis (hemorrhage)	25	Medical management
9	Foreign body (pieces of towel)	10	Surgery
10	Gastritis (ulcer or erosion, hemorrhage)	25	Medical management
11	Gastritis (hemorrhage)	40	Medical management

## Data Availability

The original contributions found in this study are included in this article. Further inquiries can be directed to the corresponding author.
